# Assessing Measurement Invariance in Allostatic Load between Black and White Adolescents

**DOI:** 10.1007/s40615-025-02817-8

**Published:** 2026-02-09

**Authors:** Abbey N. Collins, Steven J. Holochwost, Matthew G. Graham, Jody L. Lin, Vanessa V. Volpe

**Affiliations:** 1 Department of Psychology, North Carolina State University, Campus Box 7650, Raleigh, NC 27695-7650, USA; 2 Department of Psychology, Lehman College, City University of New York, New York City, USA; 3 Division of Hospital Medicine, Department of Pediatrics, Spencer Fox Eccles University of Utah School of Medicine, Salt Lake City, USA

**Keywords:** Allostatic load, Measurement invariance, Adolescents, Race, Health disparities

## Abstract

**Background:**

Allostatic load (AL) reflects the cumulative dysregulation of physiological systems due to repeated stress responses. Early life exposure to adversity can increase AL, causing premature aging and heightened mortality risks, especially among Black adolescents. AL has potential to be a useful proxy measure of health risk. However, for it to be used in racially-diverse populations, measures of AL must be invariant across racial groups, particularly when comparisons are being made between the groups.

**Purpose:**

This exploratory study examines whether AL displays properties of measurement invariance across Black and White adolescents.

**Methods:**

We used a nationally representative sample drawn from the National Health and Nutrition Examination Survey dataset to establish the optimal model for this sample. We then tested for measurement invariance by conducting a multigroup model.

**Results:**

The construct of AL displayed weak factorial invariance, such that the same biomarkers indexed AL for both groups (Likelihood Ratio Test: $\left(\chi^{2}(3)=7.02,p=.071\right)$. AL did not display strong factorial invariance, suggesting that mean comparisons are not valid between these two groups.

**Conclusion:**

These initial results underscore the importance of testing assumptions about measurement invariance before comparing levels of AL between Black and White adolescents. Research assessing AL during adolescence may help clarify researchers’ and clinicians’ understanding of the interplay between early life adversity, puberty, and stress-related biomarkers that contribute to AL. This construct may facilitate more effective identification and prediction of adolescents’ disease risk, but it remains critical to first establish how to accurately model and apply AL in racially-diverse populations.

Allostatic load is a multisystemic index of the wear-and-tear on bodily systems due to chronic mobilization of the body’s physiological systems in response to accumulative stress or disadvantage [1]. Higher allostatic load indexes biological risk [2] and is associated with a wide range of health outcomes, including increased risk of disability, pain, cardiovascular disease, cancer, diabetes, psychological distress, and mental illnesses [3–5]. However, the clinical utility of allostatic load as an index of sub- or pre-clinical disease risk is dependent on its measurement. Research has examined heterogeneity in the composition, scoring, and construct validity of allostatic load that may undermine this utility [4, 6–11]. In the current study, we add to this important body of research. As untested assumptions about the structural validity of this construct may distort conclusions about racial disparities, we take an initial step toward understanding the measurement invariance of allostatic load in Black and White adolescents.

Allostatic load is conceptualized as the chronic activation of several physiological pathways, which are indexed by biomarkers that, taken together, comprise an allostatic load score. These physiological pathways are denoted as primary mediators, primary effects, secondary outcomes, and tertiary outcomes [12–15]. *Primary mediators*, such as stress hormones (e.g., epinephrine, cortisol) and anti-inflammatory cytokines, are released in response to stress. Hormones and cytokines exert *primary effects* on tissues and organs at the cellular level by affecting enzymes, receptors, ion channels, and genes. Chronic stress can lead to the prolonged overactivation or underactivation of primary mediators as the body becomes dysregulated. Dysregulation can lead to *secondary outcomes*, which are integrated processes that reflect the cumulative effects in a specific tissue or organ in response to the primary mediators. Ultimately, dysregulation of biomarkers within these systems can lead to *tertiary outcome*s, or clinical morbidity and mortality. There is tremendous variability in the number of and specific biomarkers selected for any given investigation of allostatic load, with constraints on biomarker inclusion being largely due to the availability of biomarkers in a given dataset [9, 16, 17]. The “original ten” biomarkers from the MacArthur Aging Studies (12-h urinary cortisol, epinephrine, norepinephrine, dehydroepiandrosterone-sulfate (DHEA-S), total cholesterol (TC) to high-density lipoprotein (HDL) cholesterol, HDL cholesterol, plasma glycosylated hemoglobin (HbA1c), aggregate systolic and diastolic blood pressure (SBP and DBP), and waist-to-hip (W/H) ratio [18] are often considered optimal, however, they may be most appropriate for older populations and there is no established gold standard for biomarker inclusion [4, 6]. To be faithful to the conceptualization of allostatic load as multisystemic dysregulation, many investigators seek to include at least 10 biomarkers from at least two different physiological systems (cardiovascular, metabolic, inflammatory) [9].

Racial disparities in allostatic load are frequently examined via race-comparative analyses. Findings indicate that non-Hispanic Black individuals (hereafter referred to as Black) have higher allostatic load than non-Hispanic White individuals (hereafter referred to as White; see Beckie [19] for a review). Among adults, this disparity appears to persist at all ages and is not fully explained by socioeconomic status [20–22]. Race-comparative studies have contrasted allostatic load values between racial groups, generating findings such as Black adults have more advanced health deterioration than White adults [23, 24]. In line with the weathering hypothesis [21], minority stress models [25], and research on embodied inequality [26] systematic disadvantage and social inequities resultant from structural racism explain greater stress and more elevated allostatic load for Black compared to White adults [27, 28] such that racial differences in allostatic load between Black and White adults are explained by experiencing discrimination, negative emotions, and disrupted sleep [29]. In this way, high allostatic load has been studied as an important indicator of physiological dysregulation, one that is thought to explain disproportionate disease risk for Black populations. Despite this consistent body of findings, foundational assessment of the comparability of the measurement of the construct of allostatic load (i.e., measurement invariance) between Black and White individuals remains scarce, undermining the results of race-comparative studies that find evidence of racial disparities.

Race-comparative allostatic load research is prevalent but its conclusions rest on several, often untested, measurement assumptions. A first methodological assumption undergirding race-comparative research on allostatic load is that the biomarkers that comprise an allostatic load score are the same for Black and White populations (i.e., that these biomarkers exhibit configural invariance, with respect to the underlying construct of allostatic load), but there is emerging evidence to suggest that this assumption may be incorrect. For instance, race-comparative research on allostatic load with Black and White adults suggests that biomarkers that index allostatic load (e.g., triglycerides) may differ in their predictive utility and should therefore not be included at all for one group or another [15]. In another study of Black and White adults, some allostatic load biomarkers were differentially predictive of depression by racial group [30]. In this way, an examination of the comparability of the underlying biomarkers that comprise allostatic load between Black and White individuals is still needed.

A related, second methodological assumption is that a given set of biomarkers contribute in similar ways to the overarching construct of allostatic load, such that the strength of the association between allostatic load and each biomarker is the same across Black and White populations (i.e., weak factorial or metric invariance). Again, this may not be the case. For example, Howard and Sparks [22] found that there were differences in the relative strength of factor loadings of each allostatic load biomarker in one investigation with Mexican American, Black, and White adults, such that two of the three strongest biomarkers of allostatic load for Black adults particularly were inflammation biomarkers. Despite the different ways in which allostatic load may be parameterized within studies, the assumption that allostatic load measures the same biological state of dysregulation regardless of the selection of specific biomarkers is compounded when studies seek to ascertain differences in a total allostatic load score between racial groups. Indeed, directly comparing these scores requires establishing strong factorial or scalar invariance, such that the mean levels (i.e., intercept terms) for each biomarker are the same across groups. Only if we can establish strong factorial invariance in allostatic load between Black and White populations can allostatic load scores be interpreted on similar scales for these groups. However, if we cannot establish measurement invariance for these groups, then allostatic load scores cannot be interpreted similarly across them and results from studies that compare levels of allostatic load by racial group may not be accurate.

Although these methodological concerns are not inherent to the measurement of allostatic load among Black and White populations during a particular developmental period, unexamined assumptions about measurement invariance may distort our understanding of racial disparities in allostatic load burden during adolescence. A majority of investigations examine allostatic load in adulthood, yet adolescence may be a sensitive period for the measurement of biomarkers, necessitating additional investigation. For instance, Holochwost et al. [7] found that the structural validity of the allostatic load construct differs by age, and that correlations between allostatic load biomarkers were weakest during the period of adolescence, which may reflect a sensitive period of rapid biological changes corresponding to puberty and body composition. Another study of the factor structure of allostatic load among adolescents suggests that waist circumference had the highest loading of all allostatic load biomarkers [31]. However, these investigations did not consider measurement invariance in allostatic load among adolescents by race. One potential reason that measurement invariance may exist is that racial differences in adiposity [32] and pubertal development [33] during adolescence shape biomarkers [10], such that the underlying structure of allostatic load may not be the same for Black and White adolescents. Although we do not explicitly examine pubertal development in the current study, we provide an initial or exploratory step toward understanding measurement invariance of allostatic load in adolescents given that such differences may exist. If invariance is not supported, future research can seek to address these mechanisms of difference.

## The Current Study

The goal of the current study is to provide an initial examination of the measurement invariance of allostatic load for Black and White adolescents. Based on existing research with Black and White adults suggesting that the biomarkers used to index allostatic load may differ for these groups [15], we hypothesize that the measure of allostatic load will not demonstrate configural invariance by racial group among Black and White adolescents. Based on research investigating racial differences among adults [6], and racial differences in adiposity and puberty during adolescence that may impact the strength of allostatic load biomarkers [10], we hypothesize that the measure of allostatic load will not demonstrate weak factorial invariance by racial group among Black and White adolescents.

## Method

Data for the current investigation is drawn from the National Health and Nutrition Examination Survey (NHANES), which is a cross-sectional survey of health and nutritional status of the United States (US) civilian, noninstitutionalized population. Data is collected in two-year cycles and uses a complex, multistage, probability sampling design to obtain a representative sample of the US population starting with newborns and extending into older adulthood. Data from NHANES was selected for the current investigation because it is one of the few publicly available nationally representative datasets that includes biomarker data for adolescents (i.e., ages 8–19). Second, NHANES includes sampling weights, and therefore it is possible to generate estimates that are representative and generalizable to the population of the US using these data [7]. In the current study we use data from the 2009–2010 NHANES cycle since it contained the largest number of biomarkers across adolescents, which is the target age group for this investigation. Additionally, the 2009–2010 cycle includes a biomarker from the inflammatory system (i.e., c-reactive protein) which is not present in more recent cycles. It is important to note that the set of biomarkers in the NHANES dataset is restricted, such that there is limited inclusion of neuroendocrine markers (e.g., cortisol, epinephrine, DHEA-S, etc.). However, we do not believe this will affect our measure of allostatic load, as there isn’t a set standard for the exact number of biological systems that should be included in measures of allostatic load, nor is there a criterion for the exact biological markers that should be used [8].

### Participants

In the current study, we focus on Black and White adolescents ages 8 to 19 years old. Although the age ranges that define adolescence may be defined differently, the onset of puberty is often a hallmark of such definitions [34]. The average age for pubertal onset in the US is between 8 and 13 years of age for girls and between 9 and 14 years of age for boys. Black adolescents have earlier pubertal onset compared to White adolescents [35]. Therefore, we considered adolescence to begin as early as age 8. Our selected adolescent age range also allows us to retain the age bands created by NHANES, ensuring that we avoid parameter estimates that would no longer be nationally representative [36]. Our analytic sample consists of 1,181 non-Hispanic Black (40.1%) and non-Hispanic White (59.9%) adolescents ($M_{\text{age}}=13.30,\;SD_{\text{age}}=3.48$). See Table 1 for participant characteristics for the overall sample and by race.

### Measures

The National Center for Health Statistics designed and conducted data collection for NHANES. Demographics, pregnancy status, and medication use data were collected by trained interviewers as part of an in-home visit. All biomarkers were collected by trained health technicians in a mobile examination center, and all laboratory samples underwent quality control measures, like random repeat testing, to identify trends, shifts, and uncertainties in the collected data [37]. Please see Zipf et al. [38] for a more detailed description of the data collection methods used in the 2009–2010 survey cycles.

### Allostatic Load Biomarkers

Biomarkers used to index allostatic load were determined by those available in the NHANES dataset for individuals 8 to 19 years old. These included systolic and diastolic blood pressure, C-reactive protein, albumin, total cholesterol, pulse, creatinine, and waist circumference. The biomarkers represent the cardiovascular (systolic blood pressure, diastolic blood pressure, total cholesterol, pulse), inflammatory (c-reactive protein), and metabolic (albumin, creatinine, waist circumference) systems.

The first group of biomarkers were collected via measurements taken at the medical examination center. Systolic and diastolic blood pressure were collected in accordance with the American Heart Association’s Protocol [39, 40]. After a 5-minute rest, participants had their systolic and diastolic blood pressure measurements taken. Three consecutive readings were obtained 30s apart. If there were any interruptions that occurred between the three readings, a fourth reading was taken. The average of all the readings were used as an index of blood pressure. For more details see on the procedure see Ostchega et al. [41]. Pulse was collected and recorded at the same time as systolic and diastolic blood pressure.

Waist circumference was assessed by taking measurements just above the right iliac crest at the mid-axillary line [42, 43]. Two measurements were taken, but a third was made if the measurements differed by more than 1.0 cm. The mean of all measurements taken were used for analysis. Body mass index (BMI) was calculated by dividing weight in kilograms by height in meters squared and rounded to the nearest tenth [44]. BMI and waist circumference were highly intercorrelated, therefore we decided to select one indicator for inclusion in our model. We selected waist circumference for inclusion as it is the most optimal predictor of intra-abdominal fat, and is the strongest predictor of stress-related cardiometabolic morbidity risk [45, 46]. Additionally, research indicates that BMI is not an optimal measure of stress-related adiposity in adolescents [47].

The second group of biomarkers, c-reactive protein (CRP) and total cholesterol, were collected via blood samples [39]. CRP was analyzed using latex-enhanced nephelometry with high sensitivity [48] and total cholesterol was calculated by using the Hitachi 704 Analyzer and reagents from Roche/Boehringer Manneheim Diagnostics, Indianapolis, IN and then calculated using the Friedwald equation [39, 49–52].

The final group of biomarkers, creatinine and albumin, were collected via random urine samples at the mobile examination center [53]. Creatinine was calculated using an enzymatic (creatininase) reaction and a Roche/Hitachi Modular P Chemistry Analyzer [39, 54, 55], and albumin was analyzed by using a solid-phase fluorescent immunoassay [42].

### Data Analytic Plan

First, we ran descriptive statistics and correlations among the biomarkers for the whole sample and within each racial group. Next, we conducted a confirmatory factor model to establish the structure of the model that would serve as a reference for the subsequent models testing measurement invariance. For the Comparative Fit Index (CFI) and Tucker-Lewis Index (TLI) we utilized the cutoff of 0.95 established by Hu & Bentler [56] to decide whether each iteration of the model was a good fit for the data. Additionally, we examined the Root Mean Square Error of Approximation (RMSEA) and used the cutoff of 0.05 or less to define adequate model fit [56]. Sampling weights were assigned using the 2-year examination sampling weights. Additionally, parameter estimates in Mplus were adjusted to account for nesting by the primary sample unit (i.e., geographical region from where the data was collected) and to account for non-independence of observations (i.e., clusters of households). For the initial confirmatory factor model, all measured variables (i.e., systolic blood pressure, diastolic blood pressure, CRP, albumin, cholesterol, pulse, waist circumference, and creatinine) were allowed to load onto a single latent allostatic load variable. The residual variances for systolic and diastolic blood pressure were allowed to covary because of their similar measurement method (i.e., common-method variance). Only the biomarkers that had significant $R^{2}$ values were retained in the final configural model.

Our decision to model allostatic load as a unidimensional construct rather than exploring higher-order factor structures was based on previous research [57, 58]. Prior studies, including those using data from NHANES [6, 22, 31], have found support for a unidimensional construct [57]. King and colleagues [31] found that a single overarching allostatic load construct was the best fit for the data collected from adolescents participating in NHANES, such that each biomarker loaded onto a single factor, rather than as a subfactor within a second-order factor structure [31]. Howard & Sparks [6] also found support for a unidimensional factor structure using data from NHANES collected from adults 25 years old or older. They noted that when attempting to model two- and three-factor solutions, there was an increase in Heywood cases (i.e., negative variance estimates for one or more factors).

Next, we examined whether our final confirmatory model displayed properties of measurement invariance between Black and White adolescents by estimating a series of multiple-group models where race (coded 0 = White and 1 = Black) was the categorical grouping variable. To do this, we utilized the CONFIGURAL, METRIC, SCALAR command in Mplus. In the configural model, all factor loadings and intercepts were free to vary across the two groups [59]. Thus, whereas the final confirmatory model sought to model the covariance structure $\Sigma_{xx}$ among the measured variables such that:

$$\sum_{xx}\;=\Lambda_{x}\Phi \Lambda_{x}^{\prime }+\theta_{\delta }$$

(where $\Lambda_{x}$ corresponds to the matrix of factor loadings, $\theta_{\delta }$ corresponds the matrix of error or residual terms, and Φ corresponds to the matrix of covariances among the latent variables, which in this case is a constant because there is only one latent variable), the configural model allowed the matrices of factor loadings and residual terms to vary by group g. Therefore, the formula for the covariance structure became:

$$\sum_{xx}^{\left(g\right)}\;=\Lambda_{x}^{\left(g\right)}\Phi^{\left(g\right)}{\Lambda \prime }_{x}^{\left(g\right)}+\theta_{\delta }^{\left(g\right)}$$

Next, we tested for weak factorial invariance (which is also referred to as metric factorial invariance) to determine whether each biomarker contributed equally to allostatic load across groups. At this step, factor loadings were fixed across both groups [59], but intercepts were still allowed to vary by group. Using the likelihood ratio test, we examined whether imposing constraints on the factor loadings led to a significant decrement in model fit. A non-significant test would indicate that we could establish weak factorial or metric invariance and would recommend testing for strong factorial or scalar invariance [59]. To do so, we constrained the intercepts to be the same across groups [59]. Establishing strong factorial invariance would indicate that each of the biomarkers contributed equally to allostatic load *and* that the mean levels of those biomarkers were equal across both groups, thus allowing for a direct race comparison in levels of allostatic load.

Research indicates that in studies of measurement invariance, statistical power does not just depend on sample size, but also factor determination and item communality [60, 61]. As a general rule, power will be adequate when the sample size is in the range of 100 to 200, communalities are above 0.70, and factors are well determined, which is defined as a limited number of factors with sufficient content validity and three to seven indicators per factor [62]. Additionally, research has noted that groups that have a sample size of at least 200 will likely have sufficient power to detect measurement invariance [61]. Although our communalities fall below the threshold of 0.70 ([0.08, 0.42]), the fact that we are testing a unidimensional factor model with a small number of indicators, together with our sample size, suggests that we have sufficient power to conduct our analyses [62]. These analyses were conducted in MPlus v7.0 using robust maximum likelihood estimation to account for missing observations on the dependent variables [63, 64].

## Results

### Descriptive Statistics

Table 2 displays descriptives and correlations for the selected biomarkers. In the overall sample, there was a moderate positive correlation between systolic and diastolic blood pressure (r=.28,p<.001), which further supported the decision to allow them to covary. There was also a moderate positive correlation between systolic blood pressure and BMI ($r=\left[0.36,0.43\right],\;p<.001$) and waist circumference ($r=\left[0.37,0.45\right],p<.001$). We see a similar pattern for the association between diastolic blood pressure and BMI (r=[0.12,0.23],p<.05) and waist circumference (r=[-0.13,0.26],p<.01). There is also a strong small to moderate positive correlation between waist circumference and creatinine (r=[0.21,0.25],p<.001).

For Black adolescents, systolic blood pressure showed a moderate positive correlation with diastolic blood pressure (r=.24,p<.001), BMI (r=.36,p<.001), and waist circumference (r=.37,p<.001), and waist circumference (r=.37,p<.001), which is similar to the associations for White adolescents for systolic and diastolic blood pressure (r=.30,p<.001), BMI (r=.43,p<.001), and waist circumference (r=.45,p<.001). Among Black adolescents, albumin had a small negative correlation with BMI (r=-.06,p<.001), waist circumference (r=-.07,p<.001), and creatinine (r=-.06,p<.05), which was comparable to the associations observed among White adolescents: albumin and BMI (r=-.06,p<.01), waist circumference (r=-.05,p<.05), and creatinine (r=.10,p<.01). There was a moderate positive correlation between waist circumference and creatinine for both Black adolescents (r=.23,p<.001) and White adolescents (r=.21,p<.001).

There was also evidence of differential associations among biomarkers between Black and White adolescents. Among Black adolescents, diastolic blood pressure showed a small negative correlation with waist circumference (r=-.13,p<.01), whereas for White adolescents there was a moderate positive correlation (r=.26,p<.001). Additionally, there was a moderate positive correlation between c-reactive protein and BMI (r=.23,p<.001) for Black adolescents but there was no association (r=.06,p=n.s.)^1^ for White adolescents. Among Black adolescents there was also a small positive correlation between c-reactive protein and pulse (r=.15,p<.05), but no such association for White adolescents (r=.04,p=n.s.). Cholesterol was associated with pulse for White adolescents (r=.15,p<.001), but not among Black adolescents (r=.08,p=n.s.).

### Measurement Model

The fit indices indicated poor fit to the data when all measured variables were allowed to load on our latent allostatic load variable $\left(X^{2}(19)=63.79,p<.001\right.$, TLI = 0.79, CFI = 0.86). In particular, neither the Tucker-Lewis Index (TLI) or the confirmatory fit index (CFI) met the threshold of 0.95 [54]. Looking at the specific biomarkers, we noted that albumin (*λ*^2^ = 0.002, *p* = .437), c-reactive protein (λ=0.009,p=.481), cholesterol (λ=0.000,p=.921), and pulse (λ=0.020,p=.278) were not significantly associated with allostatic load, and that allostatic load did not contribute to a significant proportion of variance in any of these variables ($R^{2}<0.01$). Therefore, our revised measurement model included only systolic blood pressure, diastolic blood pressure, waist circumference, and creatinine. This model was a good fit to the data ($\chi^{2}(1)=1.50,p=.220$, CFI=0.99, TLI = 0.98, RMSEA^3^ = 0.02)—each biomarker was significantly associated with allostatic load—systolic blood pressure (λ=0.265,p<.001), diastolic blood pressure (λ=0.265,p<.001), waist circumference (λ=0.672,p<.001), and creatinine (λ=0.092,p=.001)—and allostatic load accounted for a significant proportion of variance in each of the biomarkers. See Table 3 for the parameter estimates for the final model.

### Measurement Invariance Analyses

We next tested for measurement invariance in allostatic load among Black and White adolescents. The results suggested that the configural model was a good fit to the data ($\chi^{2}(2)=1.66,p=.436$, CFI = 1.00, TLI = 1.00, RMESA =0.000). The likelihood ratio test comparing the relative fit of the configural and metric models was not significant ($\chi^{2}(3)=7.02,p=.071$), indicating that imposing constraints to make the factor loadings equal across the groups did not lead to a significant decrement in model fit. In contrast, the results of the likelihood ratio test comparing the metric and scalar models was significant $\left(\chi^{2}(7)=54.66\right.,\;p<.001$), indicating that imposing constraints to make the intercepts equal across groups did lead to a significant decrement in model fit. These results indicated that while the same biomarkers contributed to allostatic load for Black and White adolescents and that these biomarkers contribute to allostatic load to similar degrees for Black and White adolescents, the levels of those biomarkers differed across groups. See Table 4 for the constraints imposed across each step of measurement invariance for each racial group and the results for each model. Additionally, Table 5 provides a summary of the results from the configural, metric, and scalar models.

### Post-Hoc Analyses

**Fig. 1:**
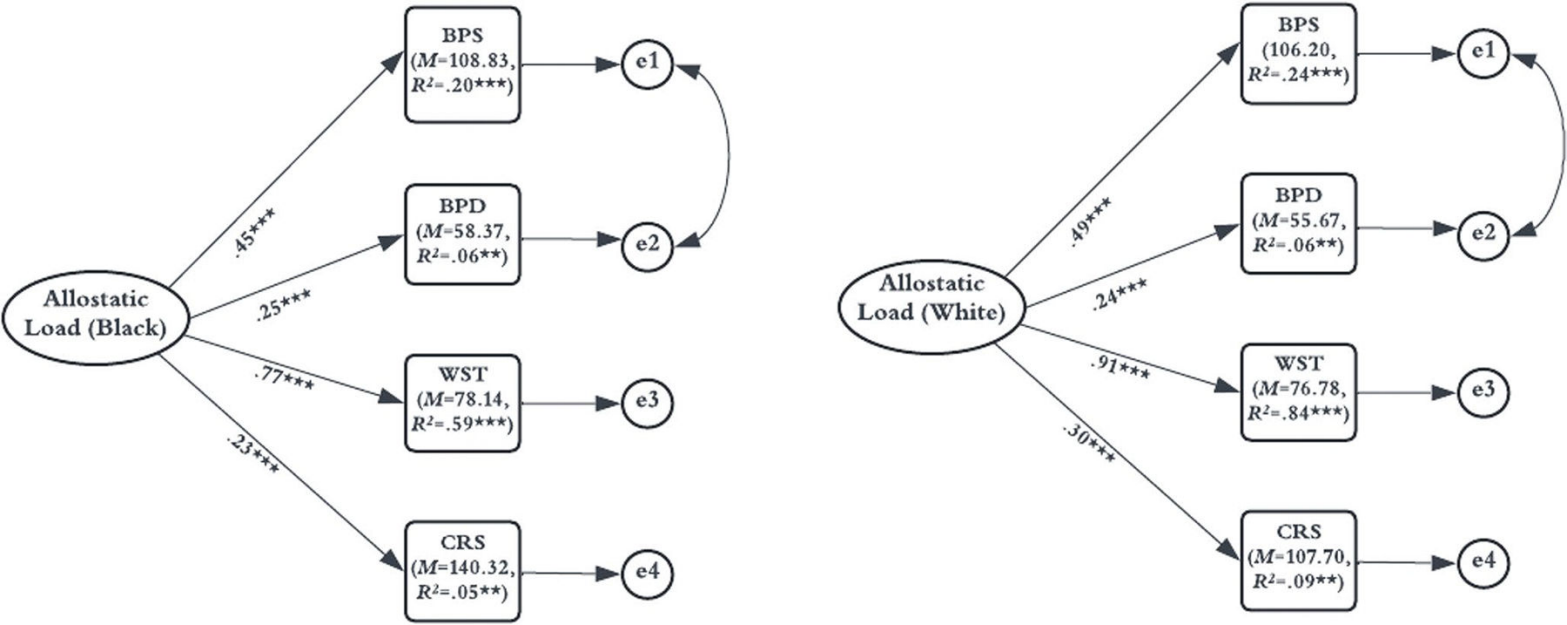
Final allostatic load model for Black and White adolescents. All factor loadings are fully standardized. Residual error terms for systolic and diastolic blood pressure have been allowed to covary. BPS = systolic blood pressure; BPD = diastolic blood pressure; WST = waist circumference; CRS = creatinine; M = Mean level of biomarkers; $R^{2}$ = R-square values. **p<.01, ***p<.001

Figure 1 shows the patterns of factor loadings and $R^{2}$ values across racial groups. Results show that systolic and diastolic blood pressure do not provide differential contributions, which is supported by the coefficients and the proportions of variance accounted for by allostatic load. For systolic blood pressure, we see that the magnitude of the association is strong for both White (β=0.49,p<.001) and Black adolescents (β=0.45,p<.001). Additionally, we see that allostatic load accounts for a similar amount of variance in systolic blood pressure for Black ($R^{2}=0.20,p<.001$) and White ($R^{2}=0.24,p<.001$) adolescents. For diastolic blood pressure, the magnitude of the association is again similar for Black (β=0.25,p<.001) and White adolescents (β=0.24,p<.001) and allostatic load accounts for a similar amount of variance for Black ($R^{2}=0.06,p=.013$) and White ($R^{2}=0.06,\;p=.030$) adolescents.

Although the results of the likelihood ratio test comparing the configural and metric models was non-significant (*ns*), it did approach statistical significance. This is reflected in the differential contribution of certain biomarkers to allostatic load across groups. For example, waist circumference has a stronger association for White adolescents (β=0.91,p<.001) compared to Black adolescents (β=0.77,p<.001), and allostatic load accounts for a greater proportion of variance in waist circumference for White ($R^{2}=0.84,p<.001$) adolescents compared to Black adolescents ($R^{2}=0.59,\;p<.001$). Creatinine also has a stronger association with allostatic load among White adolescents (β=0.30,p<.001) compared to Black adolescents (β=0.23,p<.001) and allostatic load accounted for more variance in creatinine for White adolescents ($R^{2}=0.09,p=.004$) compared to Black adolescents ($R^{2}=0.05,\;p=.004$).

Our results show that while the contribution of biomarkers to allostatic load do not differ significantly across groups, the mean levels of these biomarkers do vary.^4^ As shown in Fig. 1, the mean systolic blood pressure ($M_{BPS}=108.83$ mmHg) is slightly higher for Black adolescents compared to White adolescents ($M_{BPS}=106.20\;mmHg$). Similarly, the mean diastolic blood pressure ($M_{BPD}=58.37\;mmHg$) is higher for Black adolescents compared to White adolescents ($M_{\text{BPD}}=55.67\;mmHg$). We see this same pattern for both waist circumference ($M_{\text{WST}}=78.14$) and creatinine ($M_{\text{CRS}}=\;140.32$) being higher in Black adolescents compared to White adolescents ($M_{\text{WST}}=76.78;M_{\text{CRS}}=107.70$).

In our measurement invariance analyses, we are also interested in examining the role of income as a proxy of socioeconomic status. In the US, there is a strong association between race and income due to the systematic historical and contemporary economic disenfranchisement of Black individuals ([67, 68]). Income, as an index of chronic socioeconomic disadvantage, is associated with allostatic load [21, 28], thus may help explain basal differences in allostatic load between Black and White adolescents. Please refer to Supplement A for the result of additional sensitivity analyses which consider socioeconomic status and our model’s ability to predict self-rated health.

## Discussion

The current investigation aimed to fill a gap in our understanding of differences in allostatic load by examining an underlying assumption of much existing allostatic load research: that the construct of allostatic load demonstrates measurement invariance for Black and White individuals. Our preliminary investigation specifically focused on a relatively less examined age group in the allostatic load literature—adolescents—to inform future research on health promotion efforts. Our results indicated that the biomarkers that comprise the construct of allostatic load are the same for Black and White adolescents and a number of these biomarkers make similar contributions to the construct of allostatic load for members of both of these groups. However, we also found that levels of allostatic load biomarkers differed for Black adolescents compared to White adolescents. These results suggest that conducting race-comparative allostatic load research should first demonstrate that indices of allostatic load display properties of measurement invariance across racial groups.

Contrary to our first hypothesis, we found support for weak measurement invariance, suggesting that the same biomarkers contribute to the construct of allostatic load for both Black and White adolescents. Although some researchers have suggested that the biomarkers for allostatic load may differ for Black and White individuals because of differences in predictive validity [15], this work has been primarily conducted with adults and has not empirically examined weak measurement invariance for the construct of allostatic load by race. It may be that predictive utility differences in biomarkers for Black and White individuals are a function of differences in biomarker levels, rather than the composition of allostatic load itself. Weak measurement invariance may suggest that the general conceptualization of the allostatic load construct is relatively robust for the general population, irrespective of race. Indeed, there is no evidence to support *innate* (i.e., compared to those resultant from structural racism) biophysiological system differences between racial groups [69, 70].

Another reason we may have been able to establish weak measurement invariance is because there may be developmental differences in the biomarkers that evidence race-related differences, such that for adolescents, biomarkers do not reflect these differences. It may be that biomarkers included in studies of adults, such as those of immune function, are more likely to evidence race-related differences resulting from structural disadvantage later in life [21]. The set of biomarkers in the current study might work well to index allostatic load across racially-defined groups in adolescence, but work less well in adulthood, as physical and physiological systems consolidate against a background of diverging life experiences [31]. For example, studies conducted with adult samples identify higher factor loadings with biomarkers from cardiovascular and inflammatory system [71, 72], potentially due to the fact that it takes more time for dysregulation to occur within these particular systems. This is why biomarkers from the metabolic system (e.g., creatinine and waist circumference) are often included for adolescent populations because they are more sensitive to stress responses during this developmental period [73, 74]. These possibilities should be examined in future research.

However, these possibilities must be weighed against the fact that although the likelihood ratio test comparing the configural and metric models yielded a result that approached significance. This reflects the fact that while the associations between some biomarkers and allostatic load were very consistent across Black and White adolescents (e.g., systolic and diastolic blood pressure), they were more variable for other biomarkers (e.g., waist circumference and creatinine). One possibility is that certain biomarkers may be relatively invariant in their respective contributions to allostatic load during adolescence among Black and White children, whereas others are relatively variant. However, as adolescents transition to early adulthood, the relative invariance of biomarkers may shift to reflect physiological changes and the impact of divergent life experiences on those changes. Another possibility, in line with conceptualizations of weathering hypothesis [21], minority stress models [25], and research on embodied inequality [26] is that the structural racism that Black adolescents experience creates fundamentally different physiological environments that may alter how stress manifests biologically and that such differences may particularly accrue in magnitude and detectability over time.

In partial support of our second hypothesis, the biomarkers contributed equally to the construct of allostatic load but differed in their mean levels for Black and White adolescents. Blood pressure, waist circumference, and creatinine biomarkers were each higher for Black compared to White adolescents, potentially due to racial group differences in the timing of pubertal maturation [32] whereby a larger proportion of Black adolescents in our study population were post-pubertal. Puberty can affect certain biomarkers [10]; for example, puberty changes metabolic processes, which can affect creatinine, and rapidly change body size, including waist circumference [32, 75]. Black adolescents are more likely to experience early life adversity and structural disadvantage such as adverse childhood experiences [76–78], racial discrimination [78], and poverty [79] as a function of disenfranchisement and minoritization, compared to White adolescents. Not only can these differential experiences result in an earlier onset of puberty for Black adolescents [80–82], affecting allostatic load, but chronic disadvantage can reciprocally affect physiological responses to stress and allostatic load levels [21, 25, 26, 79, 83]. Both of these experiences could have led to higher basal levels of the biomarkers contributing to allostatic load in our sample for Black adolescents. Although we were unable to examine if pubertal timing or environmental exposure differences contributed to our failure to examine strong factorial invariance, future research should examine these possibilities.

We may have seen higher levels of allostatic load biomarkers for Black adolescents when compared to White adolescents because of group differences in socioeconomic status. In this study, there was a difference in income-to-needs ratio across groups, with Black adolescents having a lower socioeconomic status compared to White adolescents (see Supplement). In the US, there is a strong association between income and race as a function of the systematic historical and contemporary economic disenfranchisement of Black individuals [82]. As inequitable socioeconomic status is a fundamental cause of racial health disparities [84] that can be proxied by allostatic load biomarkers, it stands to reason that socioeconomic status differences may help explain basal differences in allostatic load between Black and White adolescents. In general, lower socioeconomic status has been associated with higher physiological dysregulation [16], as cumulative disadvantage serves as a chronic stressor and impacts the material conditions associated with individuals’ health such as health insurance status, access to nutrition and physical activity resources, and neighborhood safety [21, 25, 26, 85–87].

### Limitations and Future Directions

Although the current study has notable strengths, it is not without limitations. One limitation is that our choice of biomarkers was constrained by the data available in the NHANES dataset. This dataset contains many secondary outcomes (e.g., waist circumference, BMI, systolic blood pressure, diastolic blood pressure) but few primary mediators (e.g., cortisol, epinephrine, norepinephrine) and does not contain biomarkers related to the neuroendocrine system [1]. Nonetheless, NHANES remains a good choice for research on adolescents, as it contains a sufficient number of biomarkers for this population. Another limitation is that we could not fully account for constructs related to race that may impact levels of allostatic load due to limitations in the measures available. Although we were able to account for socioeconomic status, measures of many other relevant constructs, including pubertal timing (which varies by race) and racial discrimination are not contained in NHANES. Additionally, because this study was most concerned about assessing measurement invariance in the construct of allostatic load, we did not account for within-group differences that may also affect allostatic load, such as differences in geographical location, socioeconomic status, adverse childhood experiences, and engagement in health behaviors. Therefore, the next step may be to assess measurement invariance in allostatic load within racial groups by additional characteristics, because each group is not homogenous [88]. Considering how race intersects with socioeconomic status, gender, and geography can better inform prevention and intervention tailoring.

Other nationally-representative, publicly-available data sets do include measures of constructs that may impinge upon allostatic load. For example, the National Longitudinal Study of Adolescent to Adult Health (Add Health) includes measures of pubertal timing, and the most recent wave of measures administered to participants in that study (Wave VI) includes indices of racial discrimination. However, biomarker data were not collected from Add Health participants until they enrolled in the study at age 12, and data on the specific biomarkers that would be required to replicate our results were not collected until Wave V, when participants were in their 30s. Indeed, there is currently no publicly-available data set that would allow for an investigation into measurement invariance in allostatic load among Black and White adolescents (ages 8 to 19) while accounting for constructs that are associated with allostatic load and race (e.g., pubertal timing and racial discrimination). Collecting these data and incorporating them in analyses of measurement invariance, whether by examining invariance as a function of these factors themselves or modeling their associations with a latent allostatic load construct, is therefore a clear priority for future research. More detailed work with datasets that include developmental, neuroendocrine, environmental, and health outcome measures is necessary to provide more complete evidence regarding measurement invariance in allostatic load.

### Implications and Conclusion

Taken together, our results provide preliminary evidence that making direct comparisons of allostatic load scores between racial groups may yield inaccurate conclusions because certain biomarkers may have different mean levels across Black and White adolescents. Extant research compares allostatic load levels between Black and White individuals. Our findings caution researchers against conducting race comparative allostatic load research for several reasons. First, comparing Black and White adolescents’ allostatic load levels may not beappropriate given that the underlying construct of allostatic load is comprised of the same biomarkers, but is not on the same scale. Second, even if allostatic were on the same scale–that is, even if basal rates of biomarkers were similar between racial groups–it would still be problematic to use race as an independent variable to predict allostatic load for a number of reasons. Using race as an independent variable fails to consider the actionable mechanisms by which racial inequities are perpetuated (e.g., experiences of discrimination [89]), invokes a comparison by which one group is treated as the norm [89, 90], treats Black individuals in the US as a monolithic group [88], and risks essentializing race as a biological variable or innate predictor of health [90]. In short, examining race as a predictor variable employs a reductionist perspective which has both conceptual as well as specific measurement problems. Instead, researchers could seek to identify specific factors above and beyond income that may affect allostatic load for each racial group.

Results that replicate the current study’s findings would have implications for how to better measure allostatic load to more effectively integrate it into clinical practice. However, given challenges with measurement invariance and particularly our concern that puberty being a strong contributor to differences to this invariance, we suggest that considering within-person allostatic load scores over time will be the most optimal way to contextualize health risk and engage health supports early in the life course in an equitable way for both Black and White adolescents. This approach would reduce not only problems with racial comparisons, but also avoid further essentializing racial groups by instead focusing on change in health risk via within-person rather than between-person factors. Particularly when considering the rising cases of obesity among children and the ongoing health inequities faced by different racial and ethnic groups, biomarkers comprising allostatic load may be promising ways to track a child’s overall health long-term and predict disease risk. Allostatic load could be one promising tool to help clinicians provide preventive care for children at higher risk of chronic illness by allowing clinicians to index the effects of psychosocial environmental stressors and disadvantage on the body longitudinally [11]. However, future studies should consider using longitudinal, multi-systemic biomarker data to confirm findings of this investigation and specifically examine within-person measurement invariance in allostatic load over time to begin establishing how allostatic load can be implemented into clinical practice.

This study extends research, which suggests that the role of biomarkers should be considered within specific populations (i.e., within adolescence) rather than treated as similar across age groups [10], to the context of racial groups of adolescents. Results underscore the importance of assessing within-group measurement invariance in allostatic load, which can provide more granular information on the factors that may differentially affect this construct within racial groups. Additionally, this study provides evidence that future work should incorporate longitudinal, multi-systemic biomarker data in future studies to validate the results of this investigation and assess how measurement invariance may vary over time within groups. Ultimately, clarifying how to accurately model and apply the construct of allostatic load in diverse pediatric populations may enhance clinical practice and preventative care. Particularly for Black adolescents and other minority groups who experience an elevated risk of chronic disease, allostatic load may bolster prevention and intervention efforts and contribute to advancing health equity early in the life course.

## Supplementary Material

Supplementary File 1

**Supplementary Information** The online version contains supplementary material available at https://doi.org/10.1007/s40615-025-02817-8.

## Figures and Tables

**Table 1:** Demographic characteristics of participants

	Full Sample (*n* = 1181)		Black Adolescents (*n* = 473)		White Adolescents (*n* = 708)	

Variable	Mean (SD) or Median	Percent	Mean (SD) or Median	Percent	Mean (SD) or Median	Percent
Age	13.30 (3.48)		13.35 (3.52)		13.27 (3.45)	
Sex						
Male		51.4%		49.5%		52.7%
Female		48.6%		50.5%		47.3%
Race						
Non-Hispanic Black		40.1%		100%		N/A
Non-Hispanic White		59.9%		N/A		100%
Household Income	$45,000 - $54,999		$35,000 - $44,999		$55,000 - $64,999	
Number of People in Household	4.03 (1.38)		4.04 (1.42)		4.02 (1.36)	
Average PIR Score	2.35 (1.65)		1.72 (1.36)		2.76 (1.69)	

**Table 2:** Correlations among biomarkers

Biomarker	Race	1.	2.	3.	4.	5.	6.	7.	8.	9.

1. Systolic BP	Black		0.24***	0.04	−0.05*	−0.03	0.36***	−0.24***	0.37***	0.08
	White		0.30***	−0.03	−0.004	−0.09*	0.43***	−0.10*	0.45***	0.15***
	Overall		0.28***	0.01	−0.02	−0.07*	0.41***	−0.17***	0.42***	0.14***
2. Diastolic BP	Black			0.01	0.001	−0.02	0.12*	−0.11*	−0.13**	0.03
	White			0.01	0.01	0.03	0.23***	−0.03	0.26***	0.12**
	Overall			0.01	0.004	0.01	0.18***	−0.08**	0.21***	0.10**
3. C-reactive protein	Black				−0.01	0.01	0.24**	0.15*	0.22**	0.04
	White				−0.02	0.03	0.06	0.04	0.08	0.10**
	Overall				−0.01	0.02	0.15***	0.08**	0.15***	0.08**
4. Albumin	Black					−0.07***	−0.06***	0.09	−0.07***	0.06*
	White					−0.02	−0.06*	0.04	−0.05*	0.10**
	Overall					−0.04	−0.06	0.06	−0.06	0.08**
5. Cholesterol	Black						0.02	0.08	−0.01	−0.09
	White						0.05	0.15***	0.04	0.01
	Overall						−0.03	0.13***	0.02	−0.05
6. BMI	Black							−0.07	0.96***	0.21***
	White							−0.03	0.96***	0.22**
	Overall							−0.07*	0.95***	0.24***
7. Pulse	Black								−0.09	−0.10*
	White								−0.03	−0.06
	Overall								−0.06*	−0.11***
8. Waist circumference	Black									0.21***
	White									0.25***
	Overall									0.23***
9. Creatinine	Black									
	White									
	Overall									
	N	1059	1059	959	1117	954	1140	1098	1092	1117
	Mean	106.8	56.7	0.15	32.0	159.1	22.5	77.2	76.7	118.4
	SD	11.2	14.8	0.52	138.2	28.4	6.1	12.4	16.0	74.0

* *p* <.05,

** *p* <.01,

*** *p* <.001

**Table 3:** Fit statistics for biomarkers in the final measurement model

	Full sample				Black adolescents				White adolescents			

Biomarker	β	SE	B	R^2^	β	SE	B	R^2^	β	SE	B	R^2^
Systolic blood pressure	0.52***	0.05	5.59	0.27***	0.45***	0.05	5.13	0.20***	0.48***	0.05	5.13	0.23***
Diastolic blood pressure	0.28***	0.05	4.23	0.08**	0.25***	0.05	3.52	0.06**	0.23***	0.05	3.52	0.05**
Waist circumference	0.82***	0.07	12.70	0.67***	0.78***	0.08	13.65	0.61***	0.91***	0.08	13.65	0.84***
Creatinine	0.30***	0.05	21.39	0.09**	0.23***	0.04	19.26	0.05**	0.29***	0.05	19.26	0.09**

In the final model, there were 36 cases that had missing data on the all the biomarkers, therefore they were excluded from the analyses

* *p* <.05,

** *p* <.01,

*** *p* <.001

**Table 4:** Constraints imposed across racial groups and results of each step of the measurement invariance analysis

Model	Biomarker	b	β	SE	*R* ^2^	Factor loadings	Intercepts	Residual variances	Factor means

	Black Adolescents								
Configural	BPS	3.96*	0.34*	0.79	0.52*	Free	Free	Free	Fixed at zero
	BPD	1.82*	0.13*	0.54	0.07				
	WST	17.62*	1.01*	4.56	0.23*^1^				
	CRS	16.07*	0.19*	5.07	0.03*				
Metric	BPS	5.20	0.45*	0.54	0.20*	Constrained to be equal across groups	Free	Free	Fixed at zero
	BPD	3.59	0.25*	0.81	0.06*				
	WST	13.68	0.77*	1.27	0.59*				
	CRS	19.44	0.23*	3.39	0.05*				
Scalar	BPS	5.62*	0.48*	0.55	0.23*	Constrained to be equal across groups	Constrained to be equal across groups	Free	Free
	BPD	3.97*	0.28*	0.83	0.08*				
	WST	12.81*	0.72*	1.22	0.51*				
	CRS	21.50*	0.24*	3.45	0.06*				
	White Adolescents								
Configural	BPS	5.56*	0.52*	0.56	0.27*	Free	Fixed at zero	Free	Fixed at zero
	BPD	4.57*	0.30*	0.89	0.09*				
	WST	12.70*	0.85*	1.26	0.72*				
	CRS	20.49*	0.31*	3.48	0.10*				
Metric	BPS	5.20*	0.49*	0.54	0.24*	Constrained to be equal across groups	Free	Free	Fixed at zero
	BPD	3.59*	0.24*	0.81	0.06*				
	WST	13.68*	0.91*	1.27	0.84*				
	CRS	19.44*	0.30*	3.39	0.09*				
Scalar	BPS	5.62*	0.53*	0.55	0.28*	Constrained to be equal across groups	Constrained to be equal across groups	Free	Fixed at zero
	BPD	3.97*	0.26*	0.83	0.07*				
	WST	12.81*	0.85*	1.22	0.73*				
	CRS	21.50*	0.33*	3.45	0.11*				

* *p* <.05; BPS = Systolic blood pressure, *BPD* Diastolic blood pressure, *WST* Waist Circumference, *CRS* Creatinine

1 R^2^ for WST in the configural model for Black adolescents was undefined due to a negative residual variance for WST. Farooq [65] and Newsom [66] suggest that a plausible solution is to impose an equality constraint between two parameters that have similar estimates. Therefore, we imposed equality constraints between WST and BPS because they had the most similar factor loadings to obtain an R^2^ value

**Table 5:** Comparisons of Configural, Metric, and Scalar models for allostatic load for Black and White adolescents

Construct	Comparison	Δχ2 (df)	*p*	ΔRMESA	ΔSRMR	ΔCFI	Invariant?

Allostatic Load	Metric vs. Configural	7.02 (3)	0.071	−0.008	0.015	0.030	Yes
	Scalar vs. Configural	54.66 (7)	0.000	−0.149	0.076	0.097	No
	Scalar vs. Metric	37.70 (4)	0.000	−0.141	0.061	0.067	No

*ΔRMSEA* change in root mean square error of approximation for specified model comparison; *ΔSRMR* change in standardized root mean square residual for specified model comparison; *CFI* change in comparative fit index for specified model comparison; *ΔTLI* change in Tucker-Lewis index for specified model comparison

## Data Availability

The data used in the current study came from the National Health and Nutrition Survey 2009–2010 dataset. These data are de-identified and publicly available at https://wwwn.cdc.gov/nchs/nhanes/default.aspx.

## Footnotes

1 *n.s.* = not significant.

2 *λ* (lamba) = factor loading.

4 BPS = Systolic Blood Pressure, BPD = Diastolic Blood Pressure, WST = Waist Circumference, CRS = Creatinine.
