# Supplementary material for: Assessing Measurement Invariance in Allostatic Load between Black and White Adolescents
Source: J Racial Ethn Health Disparities. Author manuscript; Available in PMC 2026 Mar 20. (PMC13004540; doi:10.1007/s40615-025-02817-8)
Supplement: Supplementary File 1 [file NIHMS2149390-supplement-Supplementary_File_1.docx]

**Assessing Measurement Invariance in Allostatic Load Between Black and White Adolescents**

Abbey N. Collins, B.A.^1^, Steven J. Holochwost, Ph.D.^2^, Matthew G. Graham, M.S.^1^, Jody L. Lin, M.D., M.S.^3^, & Vanessa V. Volpe, Ph.D.^1^

^1^Department of Psychology, North Carolina State University

^2^Department of Psychology, Lehman College, City University of New York

^3^Division of Hospital Medicine, Department of Pediatrics, Spencer Fox Eccles University of Utah School of Medicine

**Corresponding Author:**

Abbey N. Collins, Department of Psychology, North Carolina State University, Campus Box 7650, Raleigh, NC 27695-7650. Email: acollin2@ncsu.edu. Telephone number: 309-706-3723

**Journal:**

Journal of Racial and Ethnic Health Disparities

**Supplemental Appendix**

**Measures**

*Ratio of Family Income to Poverty*

In sensitivity analyses, we also considered whether invariance in allostatic load was due to differences between the groups in income. The current study uses poverty-to-income ratio (PIR) as a proxy of socioeconomic status. PIR was calculated by dividing family or individual income by the poverty guidelines specific to the survey year [92]. Additionally, this measure was specific to the state each participant resided. In the NHANES protocol, PIR was not computed if the respondent only reported income as < $20,000 or ≥ $20,000 [93]. Additionally, values at or above 5 were coded as 5 or more because of disclosure concerns. Ratios below 1 indicate that family income is below the official definition of poverty and ratios of 1 or greater indicate family income at or above the poverty level [94]. The values were not computed if the income data was missing. Scores range from “0” (*no income*) to “5” (*≥ 5 times the federal poverty level*). For our analyses, we treated this variable as continuous. However, other studies have treated this variable as categorical with PIR levels defined as low income (PIR<1), middle income (1≤PIR<4), and high income (PIR ≥ 4) [93, 95]. Therefore, we also conducted our analyses with dummy-coded variables (low, middle, high) to see if results differed when the groups we treated separately.

*Self-rated health*

Self-rated health was assessed with a single item during the in-home interview. This item was measured on a standard 5-point Likert type scale from “1” (*Excellent*) to “5” (*Poor*) [96]. For adolescents 16 years of age and older and emancipated minors, they were interviewed directly and for those under 16 years of age or who could not independently answer the questions, a parent or guardian provided answers on the participant’s behalf [43]. For the current analysis, self-rated health was treated as a nominal variable, which has been done in previous [e.g., 97, 98]. The categories were: 1) *Excellent*, 2) *Very good* and 3) *Suboptimal health (Good/Fair/Poor)*.

**Analytic Plan**

We are interested in examining the role of income as a proxy of socioeconomic status in our measurement invariance analyses, given that: 1) there is a strong association between race and income in the United States, where these data were collected (e.g., [73]), and 2) income, as an index of chronic socioeconomic disadvantage, is associated with allostatic load [21, 28]. Therefore, our sensitivity analyses investigated whether there was an association between income (as indexed by PIR) and allostatic load levels, and whether including PIR in the confirmatory measurement invariance models impacted our results.

For our sensitivity analysis, we were interested in examining whether the allostatic load index performs equally well across Black and White adolescents in predicting self-rated health. Therefore, we conducted a multinomial logistic regression in Mplus. Category 1 is *Excellent*, category 2 is *Very good*, and category 3 is *Suboptimal health*. The reference is category 3. Analyses are conducted separately for Black and White adolescents.

**Results**

We examined whether there was an association between income (as indexed by PIR) and allostatic load levels. Results showed that PIR was not significantly associated with allostatic load for the sample as a whole (*β*=-0.05, *p*=0.410) nor was it significant within Black (*β*=0.05, *p*=.150) or White (*β*=-0.09, *p*=.103) adolescent subsamples.

We were also interested in assessing whether there were significant differences in PIR between groups. To assess this, an independent samples t-test was conducted and showed that White adolescents had a higher PIR score (*M* = 2.76, *SD* = 1.69) compared to Black adolescents (*M* = 1.72, *SD* = 1.36), *t*(1045.62)= 11.26, *p* < .001, *d* = 0.67. Since previous work has also treated PIR as categorical [95, 96], we ran a contingency coefficient analysis to assess whether race (Black vs. White) was related to PIR (low, middle, high). Results indicate that there was an association between race and PIR (*χ2* (2) = 101.12, *p<* .001) and the contingency coefficient revealed that PIR was dependent on race (*C* = .28, *p*<.001), such that one’s race played a role in which PIR category they were in.

After establishing our model of allostatic load, we were interested in examining whether the allostatic load index performs equally well across Black and White adolescents in predicting self-rated health. To assess this, we conducted a multinomial logistic regression. For Black adolescents, there was significant association between allostatic load and odds of reporting excellent health compared to suboptimal health. A one-unit increase allostatic load is associated with 1.80 times higher odds of being in the excellent health group compared to the suboptimal health group (*OR* = 1.80, *p*=.010, 95% CI [1.36, 2.38]). Conversely, for White adolescents, there was a significant association between allostatic load and odds of reporting very good health compared to suboptimal health. Specifically, a one-unit increase in allostatic load was associated with 20% lower odds of reporting very good health compared to suboptimal health (*OR* = 0.80, *p*=.017, 95% CI [.67, .95]).

References

1. Minhas AMK, Jain V, Li M, et al. Family income and cardiovascular disease risk in American adults. *Sci Rep*. 2023;13(1):279. doi:10.1038/s41598-023-27474-x
2. Yi H, Li M, Dong Y, et al. Nonlinear associations between the ratio of family income to poverty and all-cause mortality among adults in NHANES study. *Sci Rep*. 2024;14(1):12018. doi:10.1038/s41598-024-63058-z
3. Kailembo A, Quiñonez C, Lopez Mitnik GV, et al. Income and wealth as correlates of socioeconomic disparity in dentist visits among adults aged 20 years and over in the United States, 2011–2014. *BMC Oral Health*. 2018;18(1):147. doi:10.1186/s12903-018-0613-4
4. Tang M, Liu M, Zhang Y, Xie R. Association of family income to poverty ratio and vibration-controlled transient elastography quantified degree of hepatic steatosis in U.S. adolescents*. Front Endocrinol*. 2023;14:1160625. doi:10.3389/fendo.2023.1160625
5. Ware JE Jr, Sherbourne CD. The MOS 36-item short-form health survey (SF-36). I. Conceptual framework and item selection. *Med Care.* 1992;30(6):473-483.
6. Bombak AE, Bruce SG. Self-rated health and ethnicity: focus on indigenous populations. *Int J Circumpolar Health.* 2012;71:18538. Published 2012 May 14. doi:10.3402/ijch.v71i0.18538
7. Cullati S, Bochatay N, Rossier C, Guessous I, Burton-Jeangros C, Courvoisier DS. Does the single-item self-rated health measure the same thing across different wordings? Construct validity study. Qual Life Res. 2020;29(9):2593-2604. doi:10.1007/s11136-020-02533-2
